# Supplementary material for: A fault-intrusion-tolerant system and deadline-aware algorithm for scheduling scientific workflow in the cloud
Source: PeerJ Comput Sci. 2021 Nov 2;7:e747. doi: 10.7717/peerj-cs.747 (PMC8576554; doi:10.7717/peerj-cs.747)
Supplement: Supplemental Information 6 [file peerj-cs-07-747-s006.zip › Myproject1.1/docs/org/cloudbus/cloudsim/power/PowerVmSelectionPolicy.html]

PowerVmSelectionPolicy


JavaScript is disabled on your browser.


- Overview
- Package
- Class
- Use
- Tree
- Deprecated
- Index
- Help

- Prev Class
- Next Class

- Frames
- No Frames

- All Classes

- Summary:
- Nested |
- Field |
- Constr |
- Method

- Detail:
- Field |
- Constr |
- Method


org.cloudbus.cloudsim.power

## Class PowerVmSelectionPolicy

- java.lang.Object
- - org.cloudbus.cloudsim.power.PowerVmSelectionPolicy

- Direct Known Subclasses:
  :   PowerVmSelectionPolicyMaximumCorrelation, PowerVmSelectionPolicyMinimumMigrationTime, PowerVmSelectionPolicyMinimumUtilization, PowerVmSelectionPolicyRandomSelection

  ---

    

  ```
  public abstract class PowerVmSelectionPolicy
  extends java.lang.Object
  ```

  The class of an abstract VM selection policy.
  If you are using any algorithms, policies or workload included in the power package, please cite
  the following paper:
  Anton Beloglazov, and Rajkumar Buyya, "Optimal Online Deterministic Algorithms and Adaptive
  Heuristics for Energy and Performance Efficient Dynamic Consolidation of Virtual Machines in
  Cloud Data Centers", Concurrency and Computation: Practice and Experience (CCPE), Volume 24,
  Issue 13, Pages: 1397-1420, John Wiley & Sons, Ltd, New York, USA, 2012

  Since:
  :   CloudSim Toolkit 3.0

- - ### Constructor Summary

    Constructors

    Constructor and Description

    `PowerVmSelectionPolicy()`
  - ### Method Summary

    Methods

    | Modifier and Type | Method and Description |
    | --- | --- |
    | `protected java.util.List<PowerVm>` | `getMigratableVms(PowerHost host)` Gets the migratable vms. |
    | `abstract Vm` | `getVmToMigrate(PowerHost host)` Gets the vms to migrate. |

    - ### Methods inherited from class java.lang.Object

      `clone, equals, finalize, getClass, hashCode, notify, notifyAll, toString, wait, wait, wait`

- - ### Constructor Detail


    - #### PowerVmSelectionPolicy

      ```
      public PowerVmSelectionPolicy()
      ```
  - ### Method Detail


    - #### getVmToMigrate

      ```
      public abstract Vm getVmToMigrate(PowerHost host)
      ```

      Gets the vms to migrate.

      Parameters:
      :   `host` - the host

      Returns:
      :   the vms to migrate


    - #### getMigratableVms

      ```
      protected java.util.List<PowerVm> getMigratableVms(PowerHost host)
      ```

      Gets the migratable vms.

      Parameters:
      :   `host` - the host

      Returns:
      :   the migratable vms


- Overview
- Package
- Class
- Use
- Tree
- Deprecated
- Index
- Help

- Prev Class
- Next Class

- Frames
- No Frames

- All Classes

- Summary:
- Nested |
- Field |
- Constr |
- Method

- Detail:
- Field |
- Constr |
- Method
